# Autograft diameter in ACL reconstruction: size does matter

**DOI:** 10.1051/sicotj/2021018

**Published:** 2021-03-22

**Authors:** Fahad N.A. Alkhalaf, Sager Hanna, Mohammed Saleh Hattab Alkhaldi, Fares Alenezi, Aliaa Khaja

**Affiliations:** Al Razi Orthopedic Hospital Kuwait

**Keywords:** ACL, Hamstring graft, Arthroscopy, Joint surgery

## Abstract

*Background*: Anterior cruciate ligament injuries are commonly seen in orthopedic surgery practice. Although anterior cruciate ligament reconstruction (ACLR) has come a long way, the causes of failure have yet to be fully understood. *Objective*: The aim of this study was to investigate whether or not the intraoperative 4-strand hamstring autograft diameter does in fact influence the failure rates of ACLR. *Methods*: Retrospective intraoperative data were collected from ACLR patients from the only tertiary center available in Kuwait. Patients who underwent ACLR from 2012 to 2018 for isolated ACL injuries were included in this study, allowing for a 24 month follow-up period The cohorts were categorized into 3 groups: patients with graft size≤8mm, 2, patients with graft sizes≥8mm with 4-strands and patients with graft sizes≥8mm with 4-strands or more. ANOVA analysis was applied to address group differences between mean graft size and strand numbers and subsequently the failure rates for each group. In addition, the Mann–Whitney *U* test was used to investigate the relationship between revision and initial ACL graft size. *Results*: Out of the 711 out of 782 patients were included in this study. Only 42.6% of the patients did not need more than 4-strands to achieve an 8mm sized autograft. The patients who had autografts≤8mm in this study accounted for 17.1% of the population. About 7.2% of these patients required revision surgery. Patients with a 4-strand autograft size that was less than 8mm were 7.2 times more at risk for ACLR failure (RR=7.2, 95% CI: 6.02; 8.35, *p*=0.007). *Conclusions*: There is a significant correlation between 4-strand autograft diameter size and the need for ACLR revision surgery.

Level of evidence: IV case series

## Introduction

Most of the patients undergo reconstruction of the ACL based on destabilizing symptoms or the urge to participate in pivoting or cutting sports [1, 2]. While the gold standard for reconstruction has always been the bone-patellar tendon-bone (BPTB). The literature has reported figures as high as 30% of BPTB patients suffering from chronic anterior knee pain [3, 4]. This is why the quadrupled hamstring tendon option has become the mainstay practice in Kuwait for anterior ACLR [3, 4]. The reason being for this trend in Kuwaiti orthopedic surgical practice is that the cultural behaviors and daily living practices would be exacerbated by the BPTB related anterior knee pain should it happen [5–7].

However, one of the downfalls of using hamstring autografts as opposed to BPTB grafts is the success of the surgery relies on larger diameter grafts [8]. Major studies have suggested that the autograft hamstring diameter should ideally range between 7 and 10mm to avoid failure [9, 10]. And the current recommendations advocate for the use of hamstring autografts with a size of 8mm or above [11–13].

Although accurate preoperative autograft size prediction is difficult, clinical studies have shown that the diameter of the hamstring autograft correlates with patient height, gender, thigh circumference and BMI [13, 14].

The size of the hamstring tendon can be accurately determined by non-invasive methods such as magnetic resonance imaging(MRI) or ultrasound imaging [9–11]. The imaging modality of choice depends on what is available in medical centers. However, ultrasound has been proven to be more accurate in predicting the autograft size in the hands of an experienced operator than an MRI scan [9–11].

In terms of ACLR and risks of deep infections, BPTB grafts have significantly lower risks of deep infections followed by hamstring tendon grafts, leaving allografts with the highest infection risk [12].

The authors of this study initially hypothesized that the Kuwaiti population generally produces smaller four-strand hamstring autografts thus accounting for the need for higher revision rates [6]. The objective of this study was to investigate the population’s general anatomical size of their intra-operative hamstring autograft and ascertain whether or not the size of the autograft influenced our ACL revision rates. Our null hypothesis was that there would be no correlation between ACLR failure rate and diameter size. The current number of the Kuwaiti population was estimated to be around 5 million people, with the Kuwaitis accounting for 20% of that number [5, 6, 15]. This study was conducted in a tertiary center with the only ACLR facility in the country. Thus, all the ACLR in the country were performed by this unit.

## Methods

The data was collected retrospectively from records held by the only ACLR unit in the country. The authors included the surgical records of patients who had isolated ACL injuries from the 1st January 2012 until the 31st of May 2018 with a minimum of 2-year follow-up required. Only patients who underwent the trans-portal anatomical reconstruction with hybrid fixation and suspensory fixation on the femur with an interference screw on the tibia [14] or the all-inside method using Arthrex^®^ equipment were included [4, 16].

Patients who had their ACLR surgeries outside of Kuwait were excluded as well as patients with incomplete operative notes, missing files, and patients with lost follow-up records. Professional or contact athletes were also excluded. Three of the authors collected the relevant information from the patient’s operative and follow-up notes. The data collected from 3 separate data forms were then cross-referenced to identify any errors or conflicting information to assure for accuracy of the results. The patients were sorted into subgroups according to autograft size, number of strands, and need for augmentation. The patients in this study were categorized into three groups; less than 8mm, 8–9mm, and more than 9mm.

To investigate the relationship between primary ACLR autograft size and the number of strands used in the surgery, three statistical methods were used. First, ANOVA was applied to find out the between-group differences of mean graft size among different numbers of strands. Next, the graft sizes were categorized into different dummy variables as the outcome and categorized the number of strands as predictors. In such a way that the relative risk (RR) of graft failure of different graft sizes for a different number of strands could be calculated. To assess the relationship between revision rates and the initial autograft, a paired t-test was performed. Chi-square tests and Mann–Whitney-*U* tests were used to assess for age as a risk factor for revision surgery.

## Results

Out of the 782 isolated ACLR cases, 71 (9.1%) of them had no record of their graft size. Out of the 711 patients included in this study, 11 patients had an ACLR revision (failure rate 2.1%), 9 of them were patients from the ≤8mm graft size group.

**Figure 1:**
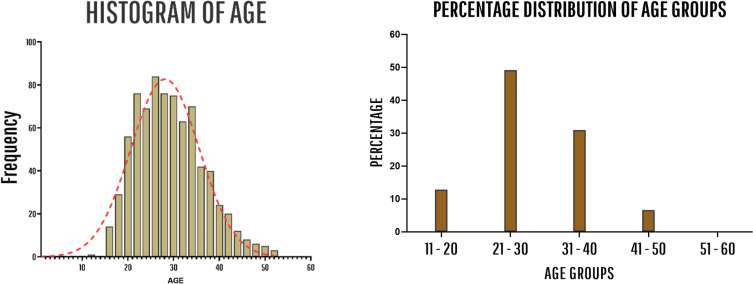
Histogram of Age and distribution plot of different age groups. Around 50% of all ACL patients were at the age between 21 and 30, while 30% of them were between 31 and 40years old.

Figure 1 and Table 1, summarize the study population demographics. The mean graft size of the sample was 8.285mm (Table 2) and graft sizes≤8mm were used in 17.1% of all surgeries. The relationship between intraoperative graft size and the number of strands can be appreciated from Table 3, the median number of strands was 4 (53.0%).

**Table 1:** Descriptive analysis of age and final graft size.

Descriptive analysis			
	Age	Final graft size (mm)	Number of strands
Mean	28.72	8.28	4.65
Median	28.00	8.00	4
Mode	22.00	8.00	4
Minimum	12.00	6.00	3
Maximum	52.00	11.00	10

**Table 2:** Percentages and Counts for different types of augmentation in different groups of graft size/strand.

Graft size/strand	Material unknown		Lars		Nylon		No augmentation		Total augmented	
	%	Count	%	Count	%	Count	%	Count	%	Count
<8mm	0.00	0	6.00	6	2.00	2	92.00	92	8.00	8
≥8 & 4-strand	0.40	1	3.21	8	1.20	3	95.18	237	4.82	12
≥8 &≠4-strand	0.00	0	2.14	5	0.43	1	97.44	228	2.56	6
Total	0.17	1	3.26	19	1.03	6	95.54	557	4.46	26

**Table 3:** Number of strands, their frequency and percentage distribution, and the mean initial graft size for different strand configurations.

Strand	Frequency	Percentage	Initial graft size	
			Mean	SD
3	2	0.3	9.500	0.710
4	379	53.0	8.056	0.630
5	266	37.4	8.317	0.589
6	29	4.1	8.614	0.723
7	11	1.6	9.400	0.690
8	12	1.8	9.125	0.744
9	11	1.5	9.250	1.060
10	1	0.2		

Table 4 shows that the quadrupled (4-strand) hamstring tendon autografts had the lowest size (8.056mm±0.63). The 4-strand hamstring tendon autograft size was significantly lower than the 5-strand (MD=−0.26±0.05, *p*<0.001), the 6-strand (MD=−0.56±0.14, *p*=0.001), the 7-strand (MD=−1.34±0.20, *p*<0.001), and the 8-strand (MD=−1.07±0.22, *p*<0.001) hamstring autografts.

**Table 4:** Post hoc test (Tukey HSD) – multiple comparisons of number of strands.

Post hoc test (Tukey HSD)						
Number of strands		Mean difference (*I*−*J*)	Std. error	*p*	95% CI	
*I*	*J*				Lower	Upper
3	4	1.4435	0.4416	**0.020**	0.137	2.750
	5	1.1826	0.4422	0.107	−0.126	2.491
	6	0.8864	0.4597	0.463	−0.474	2.247
	7	0.1000	0.4822	1.000	−1.327	1.527
	8	0.3750	0.4921	0.988	−1.081	1.831
	9	0.2500	0.6225	1.000	−1.592	2.092
4	5	−0.2609	0.0549	**0.000**	−0.423	−0.098
	6	−0.5572	0.1373	**0.001**	−0.964	−0.151
	7	−1.3435	0.2000	**0.000**	−1.935	−0.752
	8	−1.0685	0.2229	**0.000**	−1.728	−0.409
	9	−1.1935	0.4416	0.099	−2.500	0.113
5	6	−0.2963	0.1392	0.337	−0.708	0.116
	7	−1.0826	0.2013	**0.000**	−1.678	−0.487
	8	−0.8076	0.2241	**0.006**	−1.471	−0.145
	9	−0.9326	0.4422	0.349	−2.241	0.376
6	7	−0.7864	0.2374	**0.017**	−1.489	−0.084
	8	−0.5114	0.2570	0.422	−1.272	0.249
	9	−0.6364	0.4597	0.810	−1.997	0.724
7	8	0.2750	0.2953	0.967	−0.599	1.149
	9	0.1500	0.4822	1.000	−1.277	1.577
8	9	−0.1250	0.4921	1.000	−1.581	1.331

Bold value denotes more significant findings.

**Figure 2:**
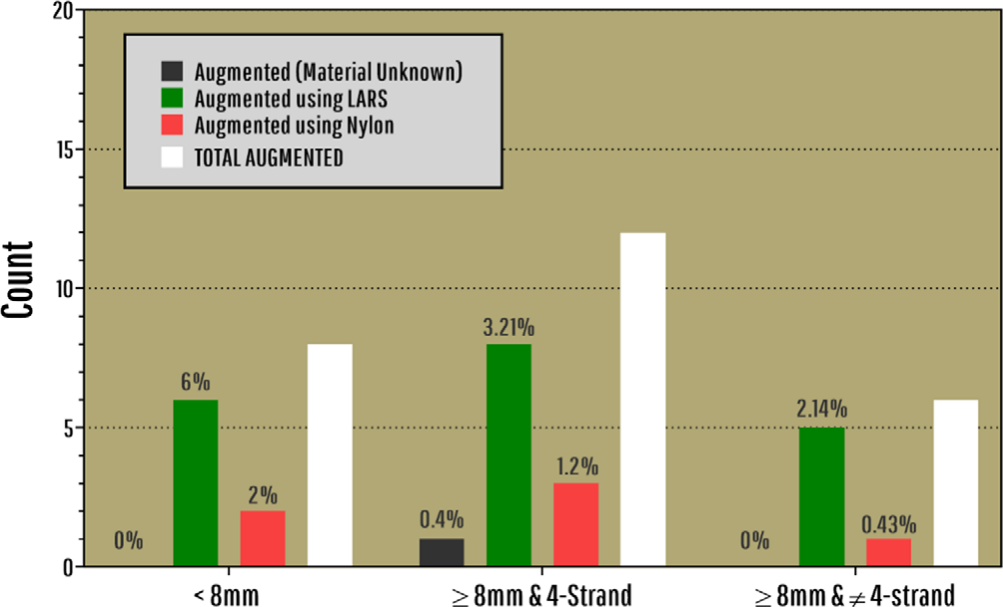
Bar Plot illustrating the number of augmentations and the materials used. In addition, the percentage of each augmentation is written on top of the bars.

According to Figure 2, a 4-strand hamstring autograft size is 1.6 times more likely to be≤8mm (RR=1.613, 95% CI: 1.46, 1.75), and this relative risk decreases as for the graft sizes≤8.5mm and≤9mm. On the other hand, the relative risk of 5-strand hamstring autograft size to be≤8mm was significantly lower (RR=0.75, 95% CI: 0.61; 0.90, *p*=0.025). This relative risk increases for graft sizes≤8.5mm (RR=0.92, 95% CI: 0.83; 1.01, *p*=0.071) and≤9mm (RR=1.03, 95% CI: 0.99; 1.06, *p*=0.083). The initial graft size of patients who did not need any revision had a statistically and significantly greater impact compared with those who underwent the revision surgery (RR=7.2, 95% CI: 6.02; 8.35, *p*=0.007). Patients who had an initial autograft size of less than 8mm, were 7.2 times more likely to need revision surgery.

**Figure 3:**
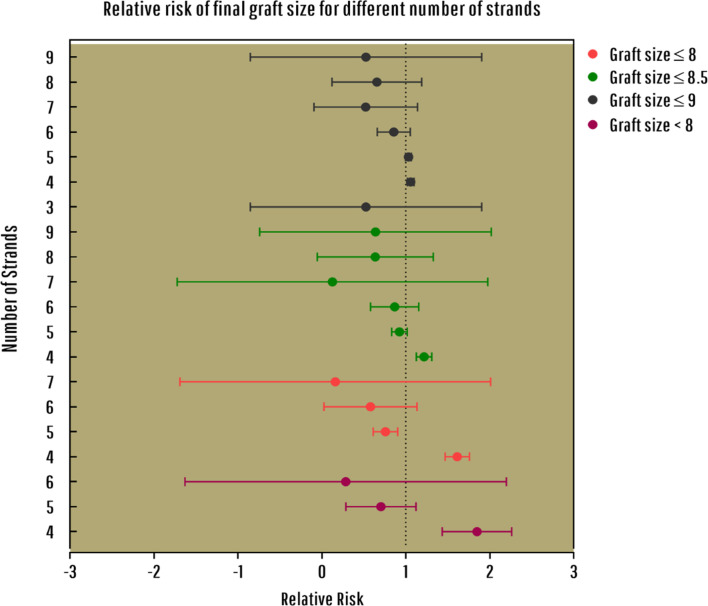
Relative risk (RR) of final graft sizes for different number of strands.

About 4.46% of all surgeries used some type of augmentation. The most common material used for augmentation was LARS^®^ with 3.26%, while only 1.03% of all autografts were augmented with Nylon (Figure 3). There was an odds ratio of 0.27 (95% CI: 0.09; 0.85) with augmentation use is 4-strand autografts that were smaller than 8mm. However, there was no statistically significant difference observed among different graft size/strand categories (*χ* ^2^(8)=8.079, *p*=0.426).

## Discussion

ACLR failure is multifactorial, the current literature agrees that the diameter size of the autograft is a major contributor to ACLR failures. There have been general ranges recommended regarding the best size for the graft [13, 16, 17]. This study’s results show a clear correlation between the hamstring autograft size smaller than 8 and the risk of revision in our study cohort. From this study, we can significantly ascertain that 4-strand hamstring autografts of at least 8mm decreases the risk of revision rates.

The Authors understand that this study has several limitations due to scarce or unavailable data regarding the study population [18]. They did not measure the posterior tibial slope, activity level, insertion site as well as intercondylar notch of each patient. These are known contributors to ACL surgery failure rates [19]. The literature has reported that females suffer from ACL ruptures in females 2–4 times more than their male counterparts, however, our sample size only had 6 females in total so they were excluded from this study [4, 19]. The authors theorize that this could be due to cultural reasons that generally discourage females from pursuing athletic careers [4–6].

In Kuwait, only 42.6% of our sample size had delivered a 4-strand 8mm diameter hamstring autograft. About 40.2% of our patients needed more than 4 strands to achieve reach 8mm±augmentation. Meaning that achieving the desired 8mm can prove to be challenging [20–22]. However, this finding is in keeping with findings from other populations, mainly in North America [13, 17, 21]. When comparing our data with the largest cohort available on hamstring graft size in ACLR, the percentage of patients that had autografts with 4-strands sized≥8mm without augmentation was also around 37.9% [13, 17, 22–24]. Whereas in South India, a similar study to ours showed that≥8mm grafts can be produced using only 3-strands in 46% of their patients [25].

While considerable success in the restoration of knee stability has been demonstrated in ACLR, recent studies indicate that between 1.8 and 22% of primary grafts will still fail globally and require revision if they are less than 8mm in diameter [25–27]. Our population results fall into the lower end (2.1%) of these internationally reported rates of revision [28–30]. From the 17.1% of patients with autografts≤8mm in our study, about 7.2% needed a revision. In the MOON study, of the 62.1% of the patients with grafts≤8mm, 15.3% needed revision [13, 14, 17]. On the other hand, the South Indian cohort had 12% of their autografts less than≤7mm and only 12% of them needed a revision [31, 32]. Table 5, is a summary of the main findings of this study compared with the large-scale studies discussed.

**Table 5:** Comparison table of results found by large-scale studies.

	Number of patients	Main findings
MOON Cohort Study	263	Revision was required in 0 of 61 patients (0.0%) with grafts greater than 8mm in diameter and 14 of 202 patients (6.5%) with 8 mm or smaller grafts (*p*=0.037)
Snaebjörnsson et al. [15]	Cases: 560	The likelihood of revision surgery for every 0.5-mm increase in the HT autograft diameter between 7.0 and 10.0mm was 0.86 (95% CI: 0.75–0.99;*p*=0.03)
	Controls: 1680	
Alkhalaf et al. (this study)	711	Patients with a 4-strand autograft size that was less than 8mm were 7.2 times more at risk for ACLR failure (RR 7.2, 95% CI: 6.02; 8.35, *p*=0.007)

Our study did not record a significant statistical difference between age and failure. However, the current evidence appearing in studies shows that revision is most common in the active and young population [33–37].

## Conclusion

Although the causes for ACLR failure rates can be attributed to multiple confounding factors, this study concluded that there is a high association between the autograft diameter size and the need for revision surgery. The 4-strand hamstring autograft diameter of less than 8mm does correlate with an increased risk of ACLR failure rates. This study shows that patients who had an ACLR with a 4-strand autograft size of less than 8mm were 7.2 times more likely to require a revision.

## Source of funding

The authors received no specific funding for this work.

## Ethical approval

The Kuwaiti Ministry of Health Ethical Committee is the main authority of patient record keeping, and they had approved this study. Committee Reference Number: 2019/1069.

## Conflicts of interest

The authors declare they have no conflicts of interest in relation to this article.
